# Effect of Uniaxial Tensile Cyclic Loading Regimes on Matrix Organization and Tenogenic Differentiation of Adipose-Derived Stem Cells Encapsulated within 3D Collagen Scaffolds

**DOI:** 10.1155/2017/6072406

**Published:** 2017-12-11

**Authors:** Gayathri Subramanian, Alexander Stasuk, Mostafa Elsaadany, Eda Yildirim-Ayan

**Affiliations:** ^1^Department of Bioengineering, University of Toledo, Toledo, OH 43606, USA; ^2^Department of Orthopedic Surgery, University of Toledo Health Sciences Campus, Toledo, OH 43614, USA

## Abstract

Adipose-derived mesenchymal stem cells have become a popular cell choice for tendon repair strategies due to their relative abundance, ease of isolation, and ability to differentiate into tenocytes. In this study, we investigated the solo effect of different uniaxial tensile strains and loading frequencies on the matrix directionality and tenogenic differentiation of adipose-derived stem cells encapsulated within three-dimensional collagen scaffolds. Samples loaded at 0%, 2%, 4%, and 6% strains and 0.1 Hz and 1 Hz frequencies for 2 hours/day over a 7-day period using a custom-built uniaxial tensile strain bioreactor were characterized in terms of matrix organization, cell viability, and musculoskeletal gene expression profiles. The results displayed that the collagen fibers of the loaded samples exhibited increased matrix directionality with an increase in strain values. Gene expression analyses demonstrated that ASC-encapsulated collagen scaffolds loaded at 2% strain and 0.1 Hz frequency showed significant increases in extracellular matrix genes and tenogenic differentiation markers. Importantly, no cross-differentiation potential to osteogenic, chondrogenic, and myogenic lineages was observed at 2% strain and 0.1 Hz frequency loading condition. Thus, 2% strain and 0.1 Hz frequency were identified as the appropriate mechanical loading regime to induce tenogenic differentiation of adipose-derived stem cells cultured in a three-dimensional environment.

## 1. Introduction

More than 32 million acute and chronic tendon injuries are reported annually in the United States, at an estimated treatment cost of $30 billion per year [1, 2]. Native tendons have a limited capacity for self-healing when injured, owing to the hypocellular nature of the tissue, with the tenocytes constituting less than 5% of the total volume [3–5]. The tendon healing process causes scar tissue formation that is different in morphology, composition, and mechanical properties compared to healthy tendons. Low cell number and scar tissue formation lead to inadequate tissue regeneration, weak matrix structure, and compromised tissue function [6, 7]. Therefore, cell-based therapies for tendon healing are paramount to augment the cell number at the repair site and aid the native healing process.

Mesenchymal stem cells (MSCs), especially bone-marrow derived stem cells (BMSCs) and adipose-derived stem cells (ASCs), are popular cell choices for tendon repair strategies due to their proliferative capacity and ability to undergo tenogenic differentiation [8–10]. Injecting autologous stem cells directly into the site of tendon repair has revealed that delivery of MSCs alone was insufficient to improve the healing. Though the cells initially showed signs of accelerating the healing process, the effect was transitional and did not result in significant differences in the tendon regeneration at long-term evaluation [11–13]. This indicated that an engineered carrier capable of sustained cell delivery in presence of appropriate chemical and mechanical cues was essential for successful tendon repairs. In the recent years, promising results have been achieved by incorporating BMSCs within tissue-engineered scaffolds, with increased tendon-related gene expression and tissue stiffness [14–16]. However, incorporating BMSCs for tendon repair strategies comes with potential complications of triggering inflammatory reactions or ectopic bone formation which would be of concern for tendon regeneration [17, 18]. Hence, nowadays, the use of ASCs for tendon tissue engineering is widely explored because of their relative abundance, ease of isolation, anti-inflammatory properties, low susceptibility to ossification, and minimal risk of donor morbidity when compared to BMSCs [19–21].

Previous studies have demonstrated that the application of cyclic mechanical loads to BMSCs cultured on elastic surfaces or within collagen matrix increases tenogenic gene expression profiles [22–24]. Mechanical stimulation is also seen to enhance tenogenic differentiation of ASCs in collagen constructs in the presence of tendon-derived extracellular matrix (ECM) [25]. However, there is no comprehensive study yet that evaluates the magnitudes of stretch and rate of loading needed for adipose-derived stem cells to commit towards a tenogenic lineage.

The objective of this *in vitro* study was to investigate the effect of different uniaxial tensile loading modalities on the matrix alignment and tenogenic differentiation of ASCs encapsulated within the three-dimensional (3D) collagen scaffolds. A custom-made uniaxial tensile strain bioreactor [26] was utilized to apply cyclic loading at 2%, 4%, and 6% strains and 0.1 Hz and 1 Hz loading frequencies to the cell-embedded collagen scaffolds over a period of seven days. Samples loaded with the aforementioned loading conditions were first evaluated in terms of viability, proliferation, and morphology of ASCs, and collagen matrix organization within the 3D scaffolds. Next, a detailed gene expression study was performed to quantify the mRNA levels of various musculoskeletal differentiation markers including ECM genes (collagens and glycosaminoglycans (GAGs)) and tenogenic, osteogenic, chondrogenic, and myogenic genes. The combined data obtained from morphological and biochemical expression analyses were used to identify the uniaxial strain magnitude and loading frequency that induce tenogenic differentiation of ASCs cultured in a 3D environment.

## 2. Materials and Methods

### 2.1. Cell Culture, Scaffold Synthesis, and Mechanical Loading Regimes

Human adipose-derived stem cells (ThermoFisher Scientific, US) were cultured in MesenPRO RS™ basal media with MesenPRO RS growth supplement (ThermoFisher Scientific, US), 200 mM glutamine (Sigma-Aldrich, US), and 1% penicillin-streptomycin solution (Gibco, US). Being the major constituent of the tendon ECM, collagen I was the preferred biomaterial to encapsulate ASCs in order to elucidate its behavior in a 3D environment [27]. Collagen I solution (Corning, US) extracted using 0.5 N acetic acid from rat tail tendons was used to synthesize the cellular 3D collagen scaffolds. Briefly, ASCs (passage 4) were encapsulated at 750,000 cells/ml seeding density within 3 mg/ml collagen I solution and neutralized to pH 7~8 with chilled 1 N NaOH solution along with PBS and cell culture media according to the manufacturer's instructions. The cell-collagen solutions were added into the loading chambers of our custom-built uniaxial tensile strain bioreactor [28] and polymerized at 37°C for 1 hour. Then, 3D cell-encapsulated collagen scaffolds were incubated in the culture media for 48 hours in a standard cell culture incubator at 37°C and 5% CO_2_.

The samples were subjected to cyclic uniaxial loading using the bioreactor at 2%, 4%, and 6% uniform linear strains at 0.1 Hz and 1 Hz loading frequencies for 2 hours/day for a period of 7 days. The strain values to mechanically stimulate the ASC-encapsulated collagen scaffolds were chosen based on the *in vivo* tendon physiology: 2% that mimics normal physiological loading (low), 4% that corresponds to intense physiological loading (medium), and 6% that induces the onset of the pathophysiological condition (high) [27]. Also, the two physiological cyclic loading frequencies selected, 0.1 Hz (low) and 1 Hz (high), correspond to gentle and rapid stretching of tendons during body movement [29]. The regular tendon rehabilitation regime of short cyclic loading (2 hours/day) for a minimum period of one week was the chosen loading duration for this study [30]. Since uniaxial tensile force governs the dynamic *in vivo* environment of tendons, it was considered to be the most relevant type of mechanical loading to stimulate ASCs towards tenogenic differentiation [27].

The samples were then harvested to characterize the cell viability, proliferation, matrix organization, and gene expression profiles of ASC-encapsulated 3D collagen scaffolds. ASC-encapsulated collagen scaffolds subjected to no loading (0%) were used as a negative control. For gene expression studies, ASC-encapsulated scaffolds cultured in media containing 1000 ng/ml BMP-12 based on a previous study [20] was used as the positive control. This was to provide a reference for direct comparison of the gene expression profile obtained due to chemical versus mechanical stimulation of ASCs within collagen scaffolds.

### 2.2. ASC Viability and Proliferation within 3D Collagen Scaffolds

The viability of ASCs encapsulated within the loaded and nonloaded 3D collagen scaffolds was examined after 7 days of loading using Live-Dead Assay kit (Life Technologies, US). The samples were incubated in 1 : 2 ratio of calcein and ethidium homodimer-1 dyes for 30 minutes at 37°C and were subsequently fixed with 4% paraformaldehyde (Sigma, US) for 30 minutes at room temperature. The cells within the scaffolds were examined using confocal microscopy at 490/525 nm and 557/576 nm excitation/emission wavelengths to visualize live (green) and dead (red) cells, respectively.

ASC proliferation within the loaded and nonloaded scaffolds was indirectly quantified by estimating the amount of DNA within each scaffold using PicoGreen ds DNA kit (ThermoFisher, US). The samples were snapped frozen in liquid nitrogen and the cells were subsequently liberated from the collagen scaffolds by mechanical disruption using a homogenizing pestle. The crushed samples were then resuspended in lysis buffer (50 mM Tris HCl, 1 mM CaCl_2_, 400 *μ*g/ml, pH = 8), and 200 *μ*g/ml of proteinase K was added to each sample solution and incubated at 55°C overnight. The lysed samples were diluted 1 : 10 in TE buffer and mixed in 1 : 1 ratio with 1 : 200 working dilution of PicoGreen dye in microplate wells. After incubating the samples at room temperature for 5 minutes, they were measured for fluorescence at 480/520 nm excitation/emission wavelengths using a microplate fluorometer (Wallac 1420). DNA in each sample was evaluated using a standard curve generated using the amounts of DNA (in ng) extracted from different cell densities of ASC and their corresponding fluorescence readings.

### 2.3. Matrix Organization within ASC-Encapsulated 3D Collagen Scaffolds

The matrix organization exhibited by the ASC-encapsulated collagen scaffolds under various mechanical loading regimes was visualized using scanning electron microscopy (SEM). After 7 days of mechanical stimulation, the loaded and nonloaded samples were fixed overnight with 4% paraformaldehyde. The samples were dehydrated by incubating them for 15 minutes each in a series of ethanol/water gradients followed by 20 minutes each in hexamethyldisilazane/ethanol gradients, both ranging from 30% to 100%. The samples were air-dried in a chemical hood overnight, sputter-coated with gold, and imaged through SEM to examine the morphology and structural changes in the loaded and nonloaded scaffolds. Further, the degree of matrix organization of the collagen fibers of each sample was quantified by obtaining directionality histograms (*n* = 4) using Fiji/ImageJ Directionality plugin (NIH, US) [31, 32].

### 2.4. Gene Expression Analysis of ASCs Encapsulated within 3D Collagen Scaffolds

The differentiation response of ASC-encapsulated 3D collagen scaffolds at various strains and loading frequencies was studied by performing expression analysis of extracellular matrix and tenogenic, osteogenic, and chondrogenic genes through quantitative real-time polymerase chain reaction (qPCR). The scaffolds were crushed, RNA was extracted using TRIzol reagent (Thermo Fisher Scientific, US), and reverse transcription was performed using Omniscript RT kit (Qiagen, US) as per the manufacturer's instructions. Quantitative real-time PCR was performed using SYBR Green PCR master mix (Thermo Fisher Scientific, US) for detecting the expression of *ECM genes* collagen I (COL I), collagen III (COL III), decorin (DCN), and aggrecan (ACAN); *tenogenic markers* tenascin-C (TCN), scleraxis (SCX), and tenomodulin (TNMD); *osteogenic markers* Runt-related transcription factor 2 (RUNX2) and alkaline phosphatase (ALP); *chondrogenic markers* Sox 9 and collagen II (COL II); and *myogenic markers* myogenic differentiation antigen (MyoD) and myogenin (MYOG), with glyceraldehyde-3-phosphate dehydrogenase (GAPDH) as the normalizing gene. The primer sequences were obtained from published literature as listed in Table 1 and purchased from Integrated DNA Technologies. PCR was performed in the iCycler iQ detection system (Biorad, US) with thermocycling performed for 40 cycles. Expression of each gene was normalized to the gene expression level of GAPDH for each sample. The data was analyzed for fold difference in gene expression with respect to the nonloaded negative control samples using the ΔΔCt method.

### 2.5. Statistical Analysis

Four samples (*n* = 4) were used for all image-based studies, and biological assays and eight samples (*n* = 8) were utilized for gene expression studies. Statistical analysis was performed by ANOVA followed by Fisher's LSD post hoc using IBM SPSS Statistics software. The data is reported as the mean and all error bars are ±standard deviation of the mean. ∗ indicates significant fold increase in loaded samples with respect to 0% nonloaded group. ∗ indicates *p* < 0.05, ∗∗ denotes *p* < 0.01, and ∗∗∗ corresponds to *p* < 0.001. † represents a significant difference between 2% and 4% groups loaded at the same magnitude of frequency while ‡ is the statistical difference with respect to 6% group loaded at the same magnitude of frequency, both with a 95% confidence interval. # represents a significant difference between 0.1 Hz and 1 Hz groups at the same magnitude of strain with *p* < 0.05. § depicts significant difference with respect to nonloaded samples chemically stimulated with BMP-12 with *p* < 0.05.

## 3. Results

### 3.1. Effect of Uniaxial Tensile Loading on ASC Viability and Proliferation within 3D Collagen Scaffolds

**Figure 1:**
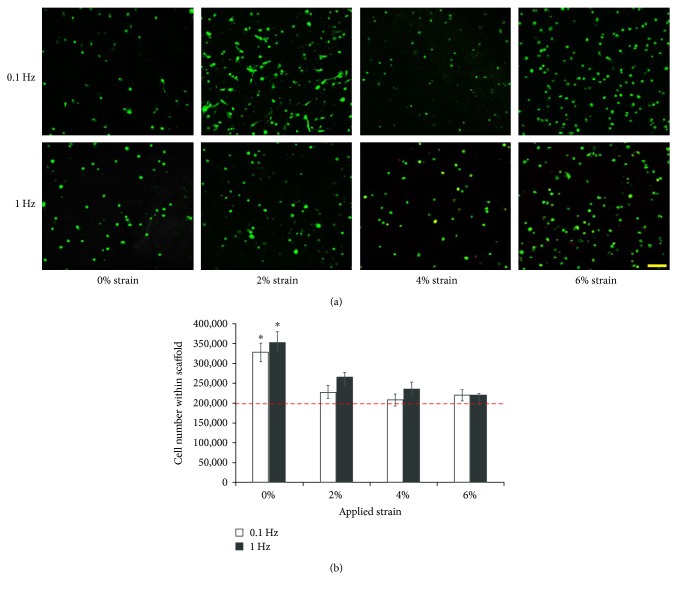
Effect of uniaxial tensile loading on ASC viability and proliferation within 3D collagen scaffolds. (a) Confocal images of ASC-seeded collagen constructs subjected to 7 days of uniaxial loading at 0%, 2%, 4%, and 6% strains and 0.1 Hz and 1 Hz frequencies. Green represents live cells and dead cells are stained red. Scale bar represents 100 *μ*m. Cells are viable within the loaded collagen scaffolds. (b) Quantification of cell number within scaffolds by estimating the DNA content. Red dotted line indicates the initial cell number in each sample. ∗ represents the statistical difference from the other groups. Cells subjected to mechanical stimulation remain viable but show limited proliferation when compared to control samples.

The effect of uniaxial tensile loading on the viability of ASCs within 3D collagen scaffolds was evaluated by performing confocal microscopy of samples stained with calcein and ethidium homodimer. The representative cell viability images for each group, namely, samples loaded at 0% (nonloaded), 2%, 4%, and 6% strains at 0.1 Hz and 1 Hz frequencies are presented in Figure 1(a).

The images indicate that the ASCs are predominantly viable (green) and no visible cell death is observed in the control and any of the loaded samples. Looking at the morphology of ASCs subjected to mechanical loading, a prominent change is evident in the sample loaded with 2% strain at 0.1 Hz frequency, where the cells are elongated, appear to have cytoplasmic extensions similar to spindle-shaped tendon cells, and are in the process of orienting themselves within the matrix. Cells from the rest of the groups including all samples loaded at 1 Hz and scaffolds loaded at 4% and 6% strain at 0.1 Hz have a rounded appearance similar to those of the nonloaded control samples.

Next, ASC proliferation within the collagen scaffolds subjected to the different uniaxial tensile loading regimes was indirectly estimated by quantifying the amount of DNA within each sample through PicoGreen dsDNA assay (Life Technologies, US) and correlating it with cell number as shown in Figure 1(b). The results confirm that there is no decrease in cell number over 7 days of culture compared to the initial density of 200,000 cells encapsulated within each scaffold indicated by the red dotted line. Further, no correlation of cell proliferation is visible with respect to both variations in strains and frequencies of the loading regimes. However, the ASCs in all the loaded groups, though viable, show limited proliferation, with the numbers ranging between 200,000 and 250,000 cells. On the other hand, a 1.5-fold increase in cell proliferation is observed within nonloaded control scaffolds (*p* < 0.05).

### 3.2. Effect of Uniaxial Tensile Loading on Matrix Organization of ASC-Encapsulated 3D Collagen Scaffolds

**Figure 2:**
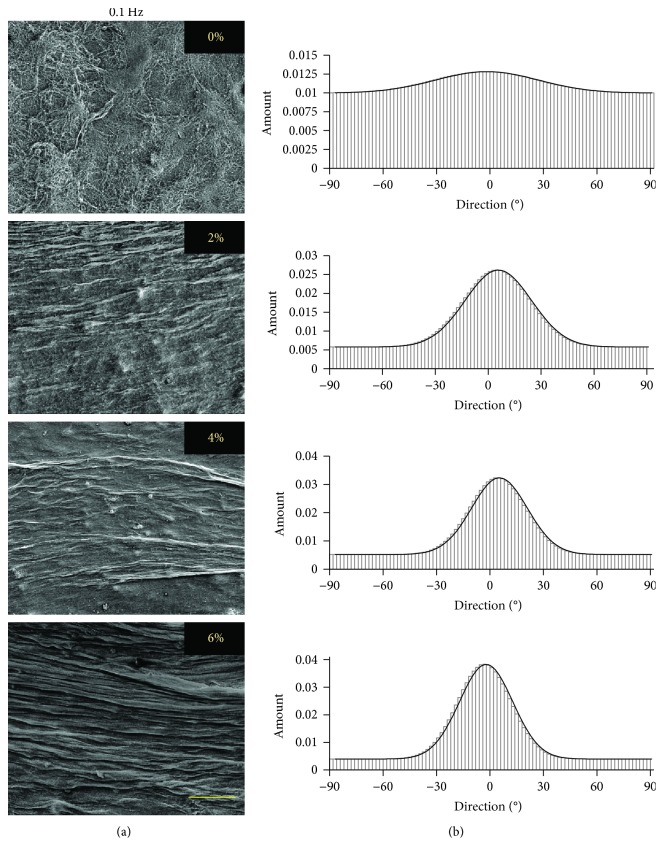
Effect of uniaxial tensile loading at 0.1 Hz frequency on matrix organization of ASC-encapsulated 3D collagen scaffolds. (a) SEM images and (b) directionality histograms of ASC-seeded collagen constructs subjected to 7 days of uniaxial loading at 0%, 2%, 4%, and 6% strains at 0.1 Hz frequency. Scale bar in the image represents 100 *μ*m. Sharper and higher peak in the histogram demonstrates a higher degree of orientation of the fibers. The matrix orientation is parallel to the axis of tensile load application.

**Figure 3:**
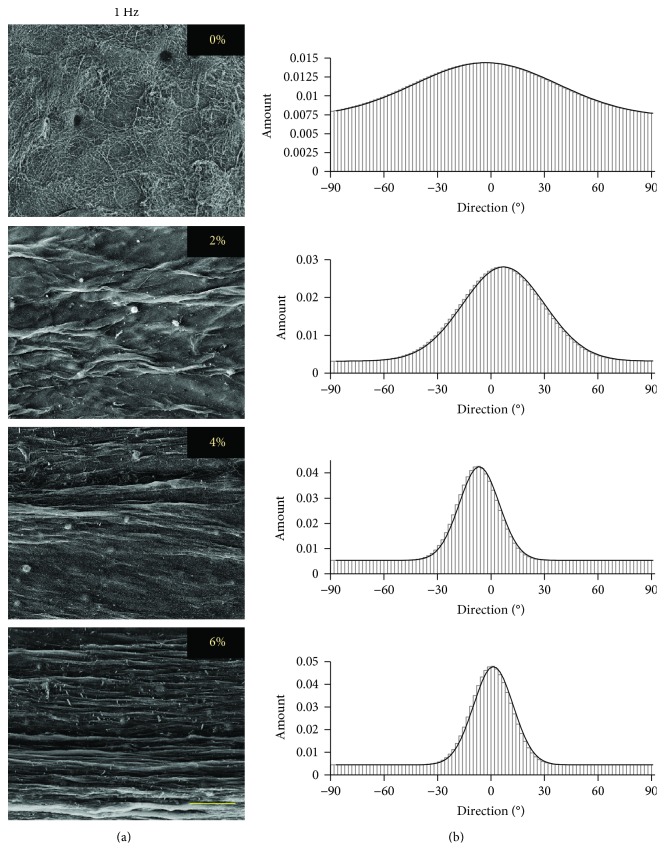
Effect of uniaxial tensile loading at 1 Hz frequency on matrix organization of ASC-encapsulated 3D collagen scaffolds. (a) SEM images and (b) directionality histograms of ASC-seeded collagen constructs subjected to 7 days of uniaxial loading at 0%, 2%, 4%, and 6% strains at 1 Hz frequency. Scale bar in the image represents 100 *μ*m. Sharper and higher peak in the histogram demonstrates a higher degree of orientation of the fibers. The matrix orientation is parallel to the axis of tensile load application.

The matrix organization induced in ASC-encapsulated collagen scaffolds due to the application of uniaxial tensile loading at different strains and frequencies was visualized through SEM. The representative SEM images of samples loaded at 0% (nonloaded), 2%, 4%, and 6% strains at 0.1 Hz and 1 Hz frequencies are shown in Figures 2(a) and 3(a), respectively.

The SEM images of control (nonloaded) scaffolds and uniaxial tensile-loaded scaffolds for both frequencies indicate that matrix organization is clearly visible in the scaffolds loaded at 2%, 4%, and 6% uniaxial tensile strains, while control scaffolds demonstrate random collagen fiber distribution. To quantify the extent of matrix organization of each sample, directionality histograms were generated using Fiji/ImageJ. The representative histograms for samples loaded at 0%, 2%, 4%, and 6% strains at 0.1 Hz and 1 Hz frequencies are presented in Figures 2(b) and 3(b), respectively. The image for the nonloaded control sample (0%) exhibits no definitive peak in the histogram indicating random distribution of collagen fibers, with directionality amount of 0.015. Significantly, the loaded samples exhibit higher directionality, with values of 0.03, 0.035, and 0.04 at 0.1 Hz and 0.03, 0.04, and 0.05 at 1 Hz, when loaded at strains of 2%, 4%, and 6%, respectively. It is also observed that the peak in each loaded sample occurs around 0° in angle, which implies that the fiber orientation of the scaffold matrix is in the direction of the uniaxial tensile load application.

**Figure 4:**
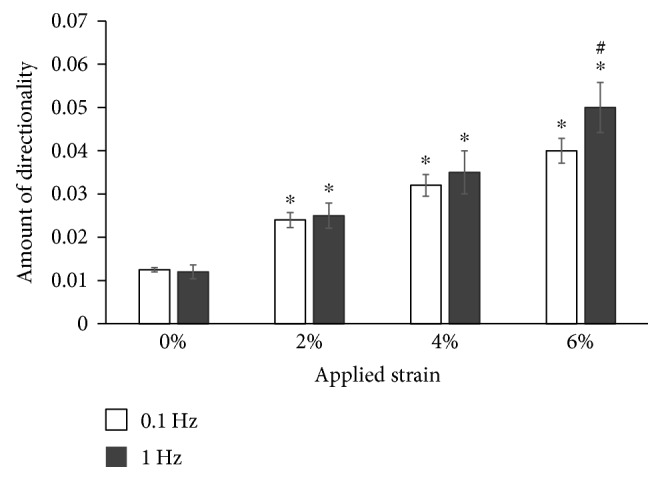
Effect of uniaxial tensile loading on matrix organization of ASC-encapsulated 3D collagen scaffolds. Quantified directionality of ASC-seeded collagen constructs subjected to 7 days of uniaxial loading at 0%, 2%, 4%, and 6% strains at 0.1 Hz and 1 Hz frequencies after consolidating the directionality histograms of SEM images obtained using ImageJ analysis shown in Figures 2 and 3. ∗ represents statistical difference from the adjacent strain group. # denotes statistical significance between same strain groups at different frequencies. The directionality of collagen fibers increases with an increase in the magnitude of strain but largely remains unaffected by the change in frequency (except for 6% group).

To determine whether the changes in the extent of matrix directionality are statistically significant, histograms obtained from images taken for each loaded group and the nonloaded control were combined and consolidated in one graph for direct comparison as shown in Figure 4.

Figure 4 demonstrates that the loaded samples at 2%, 4%, and 6% strains show statistically significant increases in their matrix alignment in comparison to nonloaded samples at both 0.1 Hz and 1 Hz loading frequencies. The applied strain values of 2%, 4%, and 6% induced a 2-fold, 3-fold, and 4-fold increase, respectively, in the amount of matrix directionality (*p* < 0.05) (Figure 4). On the other hand, no such correlation is observed with change in loading frequencies at the same applied physiological strains of 2% and 4%. Interestingly, the higher strain magnitude of 6% corresponding to the pathophysiological loading of tendon depicts a significant increase in matrix directionality with a change in frequency from 0.1 Hz to 1 Hz (*p* < 0.05).

### 3.3. Effect of Uniaxial Tensile Loading on ECM Gene Expression of ASCs Encapsulated within 3D Collagen Scaffolds

**Figure 5:**
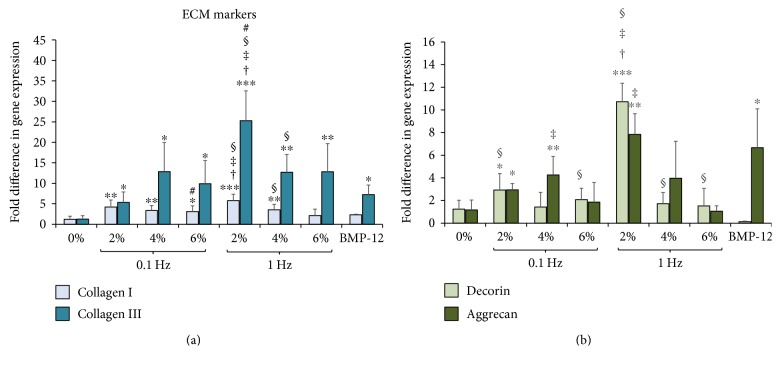
Effect of uniaxial tensile loading on ECM gene expression of ASCs encapsulated within 3D collagen scaffolds. (a) Gene expression profiles of ASCs encapsulated within collagen scaffolds subjected to BMP-12 treatment or uniaxial loading at 0%, 2%, 4%, and 6% strains at 0.1 Hz and 1 Hz. The graphs depict fold changes in various extracellular matrix genes: collagen I and collagen III, (b) GAGs: decorin and aggrecan. ∗ indicates significant fold increase with respect to the 0% samples. ∗ indicates *p* < 0.05, ∗∗ denotes *p* < 0.01, and ∗∗∗ corresponds to *p* < 0.001. † represents a significant difference between 2% and 4% groups while ‡ is the statistical difference with respect to 6% group, both with a 95% confidence interval. # represents a significant difference between 0.1 Hz and 1 Hz groups at the same magnitude of strain with *p* < 0.05. § depicts significant difference with respect to unloaded samples chemically stimulated with BMP-12 with *p* < 0.05. The mechanically stimulated samples display an increased level of ECM markers compared to the nonloaded scaffolds.

The effect of uniaxial tensile loading on the expression of ECM genes such as collagen and glycosaminoglycans (GAGs) was investigated through qPCR. PCR was performed after 7 days of mechanical stimulation at 0% (nonloaded; negative control), 2%, 4%, and 6% strains at 0.1 Hz and 1 Hz frequencies. ASC-encapsulated collagen scaffolds chemically stimulated with BMP-12 were used as the positive control.  Figure 5(a) shows the fold change in collagen I and collagen III gene expression in ASCs at 2%, 4%, and 6% uniform strains at 0.1 Hz and 1 Hz frequencies of cyclic loading and BMP-12 treated samples with respect to the 0% (nonloaded) group.

Figure 5(a) demonstrates significant increases in both collagen I and collagen III expressions in mechanically loaded samples at the aforementioned applied strains and frequencies when compared to the 0% group. Collagen III shows 5- to 25-fold increase in expression in the ASC-encapsulated samples subjected to uniaxial tensile strains of 2%, 4%, and 6% at loading frequencies of 0.1 Hz and 1 Hz in comparison to the nonloaded samples (*p* < 0.05). The chemically stimulated positive control group (BMP-12) is observed to have a 5-fold increase in collagen III expression (*p* < 0.05). Further, collagen I displays a 3- to 5-fold statistically higher expression in all ASC samples subjected to uniaxial tensile mechanical loading, including 2%, 4%, and 6% strains at 0.1 Hz and 1 Hz frequencies (*p* < 0.05), except for samples loaded at 6% strain and 1 Hz. Interestingly, BMP-12 is unable to elicit a significant increase in collagen I when compared to nonloaded scaffolds.

Figure 5(b) depicts the fold difference in GAG expression exhibited by loaded ASC-encapsulated collagen scaffolds and positive control group through studying the expression levels of decorin and aggrecan. GAGs do not exhibit a global increase in expression with uniaxial tensile loading, unlike collagens. In fact, the only groups that show predominantly significant increases in both decorin and aggrecan are the ones loaded at 2% strain at both 0.1 Hz and 1 Hz frequencies. Samples subjected to 2% at 0.1 Hz result in a 2-fold increase in decorin and aggrecan in comparison to the control (*p* < 0.05), while 2% at 1 Hz group exhibits 8–10 times increase in decorin (*p* < 0.001) and aggrecan expression (*p* < 0.01). No significant rise in GAG expression is seen in the rest of the mechanically loaded regimes except for a 3-fold increase in aggrecan at the loading condition of 4% and 0.1 Hz (*p* < 0.01). Finally, ASC-encapsulated scaffolds treated with BMP-12 do not show a change in the expression of decorin but exhibit a 5-fold increase in aggrecan expression (*p* < 0.05).

Observing the ECM results for 0.1 Hz and 1 Hz in Figure 5, it appears that among 2% strained samples, the higher frequency of 1 Hz is able to stimulate more ECM production than 0.1 Hz. However, statistical analysis between 0.1 Hz and 1 Hz groups strained at 2% reveals a significant increase only in case of collagen III expression (*p* < 0.05). Samples strained at 4% and 6% show little variation in the expression profiles of the four ECM genes between the loading frequencies of 0.1 Hz and 1 Hz.

Thus, Figure 5 reveals that (a) mechanical loading at specific loading regimes effect an increase in the expression of ECM genes of ASCs encapsulated in 3D collagen scaffolds, (b) there are clear differences in ASC response in terms of ECM stimulation with both varying strains and frequencies with respect to both nonloaded and BMP-12-treated samples, and (c) significant increases in both tendon-specific collagens and GAGs are seen for groups strained with 2% at both 0.1 Hz and 1 Hz while 4% strain at 0.1 Hz and 1 Hz stimulates significant increases in predominantly the collagen genes.

### 3.4. Effect of Uniaxial Tensile Loading on Tenogenic Differentiation of ASCs Encapsulated within 3D Collagen Scaffolds

**Figure 6:**
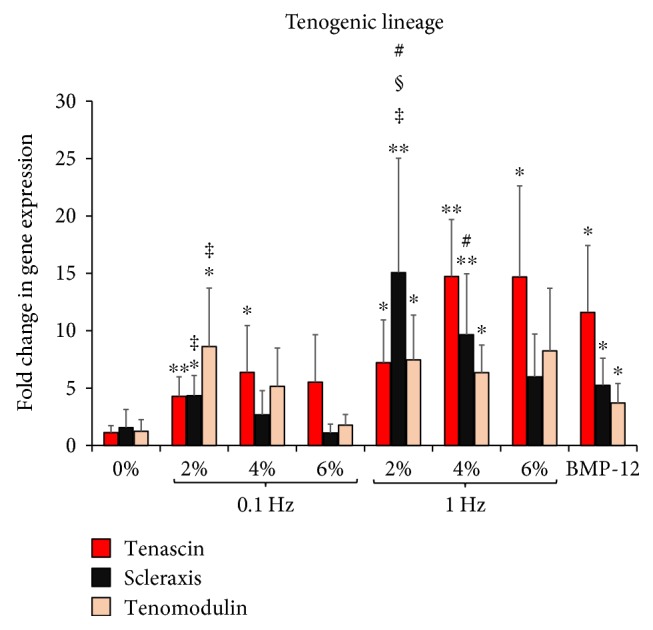
Effect of uniaxial tensile loading on tenogenic gene expression of ASCs encapsulated within 3D collagen scaffolds. Gene expression profiles of ASCs encapsulated within collagen scaffolds subjected to BMP-12 treatment or uniaxial loading at 0% (unloaded), 2%, 4%, and 6% strains at 0.1 Hz and 1 Hz loading frequencies. The graph depicts fold changes in various tenogenic markers: tenascin-C, scleraxis, and tenomodulin. ∗ indicates significant fold increase with respect to the 0% samples. ∗ indicates *p* < 0.05, ∗∗ denotes *p* < 0.01. ‡ is the statistical difference with respect to 6% group, both with a 95% confidence interval. # represents a significant difference between 0.1 Hz and 1 Hz groups at the same magnitude of strain with *p* < 0.05. § depicts significant difference with respect to unloaded samples chemically stimulated with BMP-12 with *p* < 0.05. Samples stimulated with 2% strain at both 0.1 Hz and 1 Hz show increased levels of all three tenogenic markers.

The expression level of tenogenic markers tenascin-C, scleraxis, and tenomodulin was quantified for samples loaded with 0% (nonloaded), 2%, 4%, and 6% strains at 0.1 Hz and 1 Hz loading frequencies, along with samples chemically treated with BMP-12 and presented in Figure 6.

The ASC-encapsulated scaffolds stimulated with BMP-12 meanwhile exhibit increased tenogenic response as established in previous studies [20, 37], with 10-fold rise in tenascin and 2-fold increases in scleraxis and tenomodulin. Among the mechanically stimulated groups, it is observed that 2% strain groups at both 0.1 Hz and 1 Hz display increases in tendon-related gene expression in ASCs. The 2% at 0.1 Hz group shows 4-fold increases of tenascin-C (*p* < 0.01) and scleraxis (*p* < 0.05) and 8-fold rise in tenomodulin (*p* < 0.05) while at 1 Hz tenascin and tenomodulin increase by 6-fold (*p* < 0.05) with scleraxis rising as high as 15-fold (*p* < 0.01) in comparison to the 0% (nonloaded) samples. Similar to the trend seen with the ECM gene expression, though 2% loaded samples at 1 Hz appear to have higher tenogenic gene expression compared to 0.1 Hz loaded samples, only scleraxis is statistically different between 1 Hz and 0.1 Hz at 2% loading regime (*p* < 0.05). Also showing higher ASC tenogenesis are samples subjected to 4% strain at 1 Hz, with 15, 9, and 6-fold increases in tenascin, scleraxis, and tenomodulin, respectively.

On the other hand, samples loaded at 6% strain at both 0.1 Hz and 1 Hz frequencies and 4% at 0.1 Hz do not show any marked increases in tenogenic genes. Thus, amongst the mechanically stimulated groups, the only three groups exhibiting significant fold increases in tenogenic markers were 2% at 0.1 Hz, 2% at 1 Hz, and 4% at 1 Hz (Figure 6). These samples notably coincide with the groups that also showed increased ECM gene expression in Figure 5.

### 3.5. Effect of Mechanical Loading on Osteogenic, Chondrogenic, and Myogenic Differentiation of ASCs Encapsulated within 3D Collagen Scaffolds

**Figure 7:**
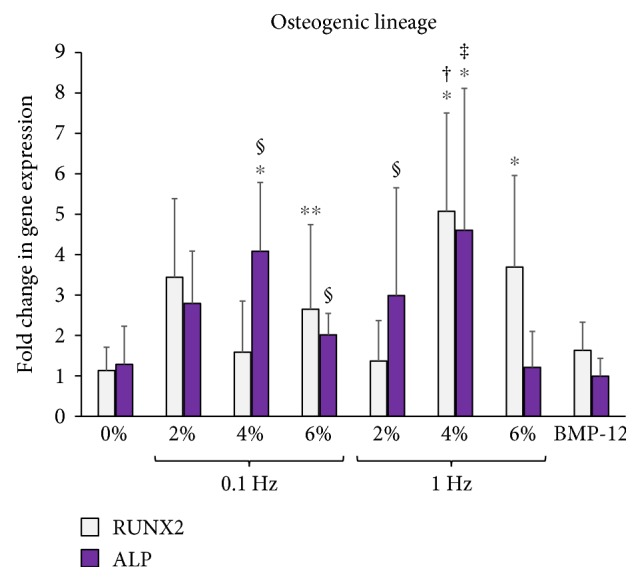
Effect of uniaxial tensile loading on osteogenic gene expression of ASCs within 3D collagen scaffolds. Gene expression profiles of ASCs encapsulated within collagen scaffolds subjected to BMP-12 treatment or uniaxial loading at 0% (unloaded), 2%, 4%, and 6% strains at 0.1 Hz and 1 Hz loading frequencies. The graphs depict fold changes in osteogenic markers: RUNX2 and ALP. ∗ indicates significant fold increase with respect to the 0% samples. ∗ indicates *p* < 0.05, ∗∗ denotes *p* < 0.01. † represents a significant difference between 2% and 4% groups while ‡ is the statistical difference with respect to 6% group, both with a 95% confidence interval. # represents a significant difference between 0.1 Hz and 1 Hz groups at the same magnitude of strain with *p* < 0.05. § depicts significant difference with respect to unloaded samples chemically stimulated with BMP-12 with *p* < 0.05. Samples stimulated with 4% strain at 1 Hz showed increased levels of both osteogenic markers.

**Figure 8:**
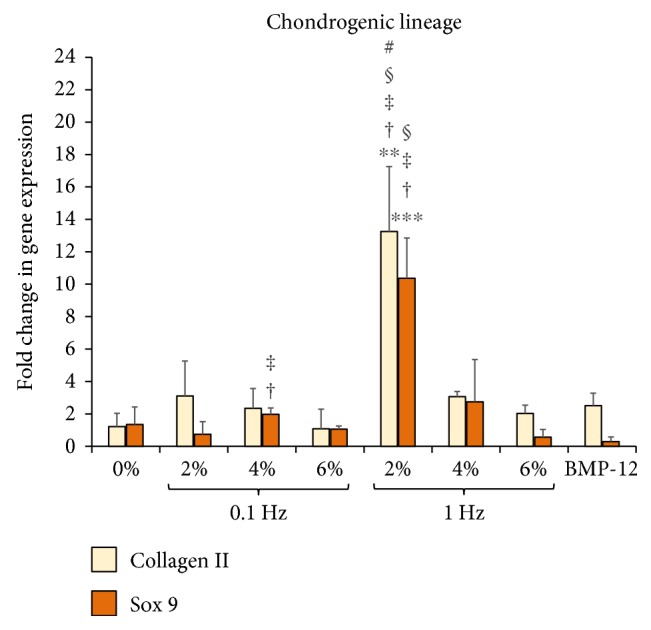
Effect of uniaxial tensile loading on chondrogenic gene expression of ASCs within 3D collagen scaffolds. Gene expression profiles of ASCs encapsulated within collagen scaffolds subjected to BMP-12 treatment or uniaxial loading at 0% (unloaded), 2%, 4%, and 6% strains at 0.1 Hz and 1 Hz loading frequencies. The graphs depict fold changes in various chondrogenic markers: collagen II and Sox 9. ∗∗ denotes *p* < 0.01 and ∗∗∗ corresponds to *p* < 0.001. † represents a significant difference between 2% and 4% groups while ‡ is the statistical difference with respect to 6% group, both with a 95% confidence interval. # represents a significant difference between 0.1 Hz and 1 Hz groups at the same magnitude of strain with *p* < 0.05. § depicts significant difference with respect to unloaded samples chemically stimulated with BMP-12 with *p* < 0.05. Samples stimulated with 2% strain at 1 Hz showed increased levels of both chondrogenic markers.

**Figure 9:**
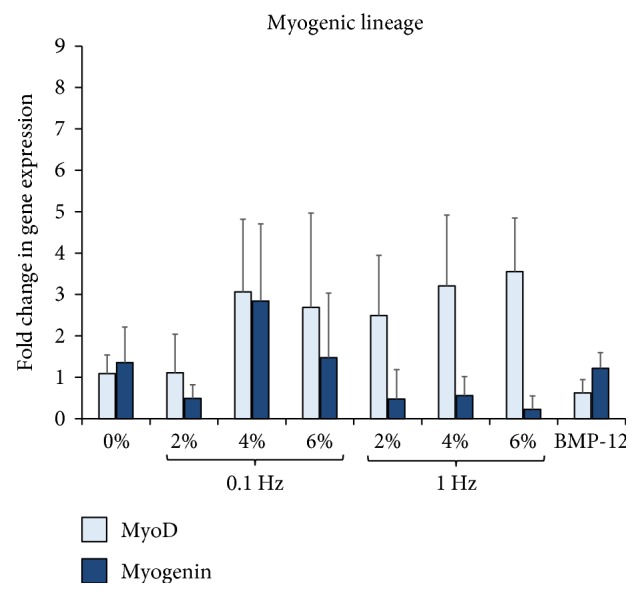
Effect of uniaxial tensile loading on myogenic gene expression of ASCs within 3D collagen scaffolds. Gene expression profiles of ASCs encapsulated within collagen scaffolds subjected to BMP-12 treatment or uniaxial loading at 0% (unloaded), 2%, 4%, and 6% strains at 0.1 Hz and 1 Hz loading frequencies. The graphs depict fold changes in various myogenic markers: MyoD and myogenin. No significant increase of myogenic markers in ASCs was observed in any of the mechanically stimulated groups.

To identify the potential for the ASCs to undergo multilineage musculoskeletal differentiation, nontenogenic markers were also evaluated. We quantified the expression levels of osteogenic, chondrogenic, and myogenic markers in ASCs encapsulated within 3D collagen scaffolds subjected to uniaxial tensile loading at 0% (nonloaded), 2%, 4%, and 6% at 0.1 Hz and 1 Hz frequencies for 7 days as shown in Figures 7, 8, and 9, respectively.

Figure 7 displays the ASC expression profile of osteogenic genes RUNX2 and ALP. Cells stimulated with the growth factor BMP-12 do not show any change in the level of osteogenic markers. Among the mechanically loaded samples, 4% strain at 1 Hz frequency is the only uniaxial tensile loading regime that exhibits increases in both osteogenic gene expression, with 4-fold increases of RUNX2 and ALP when compared to the 0% (nonloaded) group.

Figure 8 depicts the ASC expression profile of chondrogenic genes collagen II and Sox 9. The results clearly demonstrate that ASCs seeded within the collagen scaffolds undergo a chondrogenic response only when stimulated at 2% strain at 1 Hz frequency, with over 10-fold increases in both collagen II (*p* < 0.01) and Sox 9 expression (*p* < 0.001) when compared to the 0% (nonloaded) samples. The rest of the groups, including the samples treated with BMP-12, do not exhibit any increase in chondrogenic markers.

Finally, the myogenic lineage commitment potential of ASCs in response to uniaxial tensile loading for myogenic genes MyoD and myogenin is displayed in Figure 9. The data reveals that there are no significant changes observed in the levels of myogenic markers in any of the groups, and neither mechanical loading nor BMP-12 treatment was able to elicit a myogenic response from ASCs after 7 days in culture within 3D collagen scaffolds.

## 4. Discussion

ASCs have been gaining popularity over BMSCs for tendon tissue-engineering strategies in recent years due to their relative abundance, ease of isolation, and anti-inflammatory properties [19, 38]. Apart from their ability to differentiate into various mesodermal lineages in the presence of chemical factors, it is known that ASCs also can respond to mechanical stimuli by undergoing changes in their morphology and biochemical expression [25, 39, 40]. However, the effect of different mechanical loading regimes on the proliferation and differentiation of ASCs remains largely unknown. Significantly, though ASCs and BMSCs are similar in many of their characteristics, there are strong evidences that ASCs tend to respond differently to mechanical stimulation when compared to BMSCs. One such early finding revealed that mechanical loading suppressed the myogenic protein expression in ASCs, whereas others studies that used similar loading parameters enhanced myogenesis of BMSCs [41–43]. These contrasting results call for a systematic study with ASCs to investigate their morphological and differentiation response to different mechanical loading regimes. Also, most of the current literature that reports the effect of mechanical forces involves monolayer cells subjected to a single and continuous mechanical loading regime ranging up to 72 hours of duration [39, 44–47]. Thus, to the best of our knowledge, there is no study that explores the effect of different physiologically relevant cyclic uniaxial tensile loading regimes in influencing the lineage commitment and morphology of ASCs within a 3D microenvironment that would be relevant to the ongoing tissue-engineering efforts for tendon healing and regeneration. Hence, through this work, we aimed to identify the magnitude of uniaxial tensile strain and loading frequency appropriate for initiating tenogenic differentiation of ASCs cultured within 3D collagen scaffolds in response to short durations of uniaxial tensile loading on a daily basis over a period of 7 days.

ASC-encapsulated 3D collagen scaffolds loaded at 2%, 4%, or 6% strain at 0.1 Hz or 1 Hz frequency for 2 hours/day over a period of 7 days demonstrated significant differences between each loading regime, in terms of their the cell morphology, matrix organization, and cell differentiation response.

The cell morphology of ASCs within most loaded groups appeared spherical in shape, similar to those in the nonloaded samples, except for the samples subjected to 2% strain at 0.1 Hz frequency. The cells in this group displayed striking changes, notably their elongated cell structure with the presence of cytoplasmic extensions similar to spindle-shaped tendon cells, and were found to be in the process of reorienting themselves within the matrix (Figure 1(a)). Further investigation of the ASC morphological response to 2% strain and 0.1 Hz loading regime would be essential to understanding its mechanotransduction signaling mechanism over the 7-day loading period. Previous mechanistic studies have demonstrated that actin cytoskeleton remodeling directs cell realignment which occurs within 12 hours of continuous loading [48, 49].

Quantification of ASC proliferation within the mechanically stimulated scaffolds revealed that though ASCs in the loaded groups were viable, they all show limited proliferation over the period of 7 days when compared to their original seeding density (Figure 1(b)). Thus, no correlation was observed between the loading strain or frequency and the extent of cell proliferation. This result concurs with certain published articles who reported little or inhibited proliferation of ASCs, BMSCs, and tendon fibroblasts when subjected to mechanical loading. Significantly, this was accompanied with higher ECM gene expression and protein synthesis compared to the nonloaded samples. In another research, BMSCs encapsulated within a polymeric biomaterial were subjected to 10% strain at 1 Hz frequency for 3 hours/day and demonstrated no significant cell proliferation over a 21-day period, but resulted in enhanced expression of collagen I, collagen III, and tenascin markers [14]. Hence, we hypothesize that the limited proliferation of ASCs on the application of uniaxial tensile loading regimes observed in this study could be an indicator of an onset of cell differentiation.

The SEM images of loaded ASC-encapsulated scaffolds showed distinct compaction of collagen fibers at each of the applied strains and frequencies, while the nonloaded control group exhibited a highly random matrix (Figures 2(a) and 3(a)). Our previous study demonstrated that even on applying the same uniaxial tensile loading regime, the extent of collagen matrix compaction can vary significantly based on the type of cells encapsulated within the scaffold [28]. Also, higher cell densities within the scaffold are known to increase the extent of matrix compaction and increase the diameter of collagen fibers [50]. Thus, this matrix organization is attributed to a combinatorial effect of mechanical loading and ASC-mediated compaction of collagen fibers, which is also in agreement with other studies that performed mechanical loading of cell-encapsulated biomaterials [51, 52]. Furthermore, a positive correlation between the strain magnitude and the degree of organization of collagen fibers was evident at both loading frequencies of 0.1 Hz and 1 Hz (Figure 4). However, no specific trend in the scaffold matrix directionality was observed between different frequencies at the same magnitude of applied strain (Figure 4). Though there was an apparent difference in the directionality between samples loaded at 0.1 Hz and 1 Hz frequencies at 6% applied strain, a closer examination of the images used for directionality analyses revealed that it was mainly influenced by a very small region of dense matrix organization at the ends of the width of the scaffolds. This is most likely due to the increased acceleration required by the bioreactor to achieve the high strain and frequency regime.

The expression profiles of the prominent ECM genes and various mesenchymal tissue markers were quantified for ASC-encapsulated scaffolds loaded at 0% (nonloaded), 2%, 4%, and 6% strain at 0.1 Hz and 1 Hz frequencies for 7 days. Significantly, collagen I, the main constituent of the tendon ECM [27], exhibited a 3–5-fold increase in mRNA expression under all of the uniaxial tensile loading regimes with the exception of 6% at 1 Hz (Figure 5(a)). Interestingly, BMP-12 treatment was unable to elicit a significant increase in collagen I levels (Figure 5(a)). This result conforms to the previous study where BMP-12 did not induce a rise in collagen I expression within ASCs [20]. This suggests that mechanical stimulation is more effective when compared to treatment with chemical factors in its ability to direct collagen I gene expression, possibly due to the combination of cell- and mechanical loading-mediated matrix organization. Collagen III, which is secreted in the early stages of ECM synthesis [53], displayed significantly higher expression in all of the loaded ASC-encapsulated samples, with the fold increases being even higher than collagen I (Figure 5(a)). This is in accordance with many previous studies that have observed increased collagen III expression in MSCs seeded with 3D tissue-engineered scaffolds [44, 54, 55]. In fact, one study involving culture of human MSCs within collagen scaffolds noticed an increase only in collagen III level but no difference in collagen I expression upon mechanical stimulation [56]. This could imply that the collagen I microenvironment induces the ASCs to increase collagen III expression more than collagen I. Nevertheless, our results are relevant to tendon tissue-engineering applications because, in a normal tendon-healing response, collagen III is secreted initially and then later replaced with collagen I [57].

Unlike collagen, GAGs, that are known to play a role in regulating the alignment and orientation of collagen fibers [53], exhibit significant increases in expression only when loaded at 2% strain at both 0.1 Hz and 1 Hz frequencies (Figure 5(b)). Though widely associated with cartilage tissues, GAGs also are an essential component of the native tendon ECM, and hence, their increase in expression is encouraging for tendon tissue-engineering strategies. This result conforms to prior studies focusing on tenogenic differentiation of ASCs that have observed an elevation in aggrecan expression [20].

Tenogenic differentiation of ASCs due to mechanical loading was determined by quantifying the expression levels of tenascin, scleraxis, and tenomodulin. Tenascin is a protein expressed during tendon development and plays a role in increasing the tissue elasticity in response to mechanical loading [14]. Scleraxis is a transcription factor detected in tendon precursor cells and is considered to be a definitive marker for tenogenic differentiation. Tenomodulin is a regulator of cell differentiation and collagen maturation [53]. Thus, the three genes are known to play a vital role in the initiation of tenogenic differentiation of MSCs. The results obtained in Figure 6 led to the identification of three groups: 2% at 0.1 Hz, 2% at 1 Hz, and 4% at 1 Hz that showed statistically significant increases in fold expression of all three tenogenic markers. Remarkably, these groups coincided with the groups that showed increased collagens and GAG expression (Figure 5).

From the combined gene expression data for ECM and tenogenic markers, no obvious correlation could be determined between the applied strain and the fold change in gene expression within ASCs at the same loading frequency. While collagens have similar expression levels when subjected to 2%, 4%, or 6% strains, GAG expression is observed to be highest at 2% strain, beyond which there is a significant decrease at the applied strains of 4% and 6%. Tenogenic markers also show no particular trend in gene expression with increasing magnitudes of strain, though 2% strain exhibits the highest tendon-specific gene expression among the applied loads. Published literature indicates that there is no threshold for applied strains after which MSC differentiation to tenocytes is always ensured. In fact, it is observed that there is an upper limit to the applied strain beyond which the gene expression either remains constant or starts decreasing [22], which is reflected in our set of results as well.

Comparing the effect of different frequencies on the ASC gene expression profile at the same applied strain value, our results indicate that tenogenic markers demonstrate a clear increase in gene expression from 0.1 Hz to 1 Hz. In case of ECM genes, both collagen and GAG expression are similar at 0.1 Hz and 1 Hz for the applied strains of 4% and 6%. Interestingly, for 2% strain, 1 Hz shows higher ECM and gene expression when compared 0.1 Hz. Though certain monolayer cell studies have suggested that higher applied frequency results in higher folds of gene expression possibly due to the acceleration of the cell signaling cascade [58, 59], other studies have failed to identify such correlation [22, 23]. From our results, it could be broadly stated that at low magnitudes of applied strain, an increase in loading frequency is able to elicit a higher amount of gene expression from ASCs.

**Figure 10:**
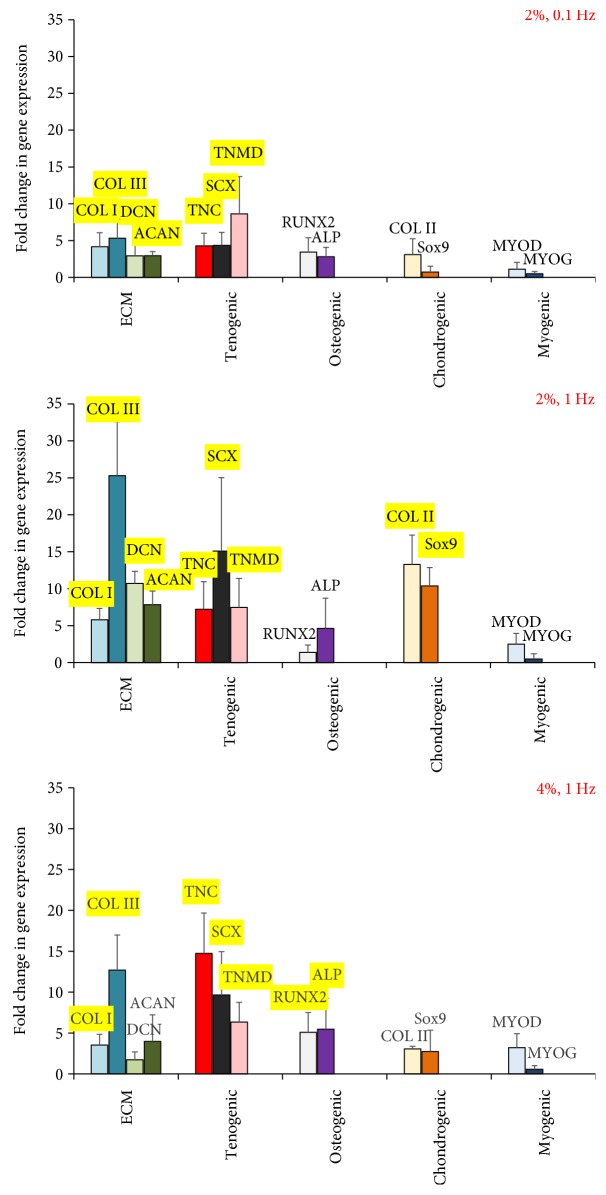
Effect of uniaxial tensile loading on ASC differentiation within collagen 3D scaffolds. Gene expression profile of ECM, tenogenic, osteogenic, chondrogenic, and myogenic markers mapped for samples loaded at 2% strain at 0.1 Hz, 2% strain at 1 Hz, and 4% strain at 1 Hz. Genes highlighted in yellow indicate statistically higher expressions when compared to nonloaded samples (*p* < 0.05).

ASCs being mesenchymal stem cells have the potential to differentiate into various musculoskeletal lineages including the bone, cartilage, and skeletal muscles in response to mechanical loading [19, 60]. For instance, an earlier study has reported the synergistic expression of bone and tendon proteins in bone marrow-derived MSCs stimulated due to mechanical loading [61]. This is undesirable for tendon tissue-engineering strategies because of the risk of the tendon getting mineralized [37]. Thus, in order to identify the appropriate uniaxial tensile strain and loading frequency for tenogenic differentiation, we also evaluated the expression levels of osteogenic, chondrogenic, and myogenic markers in mechanically stimulated ASCs encapsulated within 3D collagen scaffolds. The key findings of the overall gene expression analysis have been presented in a concise manner in Figure 10.

The previously identified groups of 2% at 0.1 Hz, 2% at 1 Hz, and 4% at 1 Hz that demonstrated significant increases in tenogenic and ECM markers were consolidated into one graph for direct comparison. Figure 10 demonstrates that the samples loaded at 2% strain and 0.1 Hz frequency display only the tenogenic differentiation markers, along with increased levels of ECM genes. The groups loaded at 2% and 4% strains at 1 Hz frequency, though exhibit increased levels of tenogenic markers, are also accompanied by elevated expression of chondrogenic and osteogenic markers, respectively. Thus, among the various uniaxial tensile loading regimes applied to stimulate the ASC-encapsulated 3D collagen scaffolds, 2% strain at 0.1 Hz frequency emerges to be the appropriate condition that is able to initiate tenogenic differentiation of ASCs, without any potential evidence of multilineage differentiation. Published studies that have investigated the effect of mechanical loading on MSCs and BMSCs have often identified the 1 Hz frequency to be suitable for tenogenic differentiation [22, 23, 62]. Even our results display equal if not higher ECM and tenogenic gene expression at 1 Hz when compared to 0.1 Hz (Figures 5 and 6). However, the risk of cross-differentiation into other musculoskeletal lineages seems to be significantly enhanced with the use of higher loading frequency of 1 Hz and hence makes 0.1 Hz the preferred choice of cycling rate when developing strategies for tendon tissue engineering. Additionally, since 2% strain and 0.1 Hz is the normal physiological loading condition for tendons, it should be appropriate for the tendon healing and rehabilitation phase.

Though the experimental design of this study was formulated and executed after thorough consideration and literature search, there exist several limitations that need to be mentioned. Firstly, since the study was designed with the aim of catering to tissue-engineering applications towards tendon healing, short duration of cyclic uniaxial tensile loading on a daily basis simulating a regular rehabilitation regime was employed, where the ASCs-encapsulated scaffolds were loaded for 2 hours/day over a 7-day duration. By applying varied combinations of low, medium, and high physiological uniaxial tensile strains and low and high loading frequencies, we identified: *(A)* the appropriate loading condition suitable for initiating tenogenic differentiation of ASCs without any cross-differentiation potential, and *(B)* a possible correlation between the magnitude of strain or frequency and the ASCs-mediated matrix organization or gene expression profiles. This, however, limited the possibility to track the morphological and functional responses of ASCs at frequent and regular intervals, which would have shed more light on the mechanistic aspect of the results. Nevertheless, having identified the most appropriate loading condition for ASC tenogenesis through this research, the next step would be to dwell deeper into the mechanisms driving the changes in ASC structure and gene expression profiles. This could be performed by including multiple time points such as day 1, 3, 5, and 7. Also, since Rho/GTPase is reported to play a major role in the transduction of mechanical strains into intracellular signals by influencing the alignment of cytoskeletal proteins like actin, using an appropriate inhibitor would allow identifying the underlying signaling mechanism governing ASCs [23, 63, 64]. Further, the correlation between the angle of ASC orientation within the matrix and the corresponding fold-changes in tenogenic expression could reveal further information regarding the mechanical signal transduction involved in the process [22].

Secondly, while the human ASCs used in this study were purchased from a commercial vendor (Thermofisher, US) and have been through rigorous quality control to meet their specifications in terms of purity, cell homogeneity, and ability to differentiate into multiple mesenchymal lineages to ensure data reproducibility, there could still be some batch-to-batch variations in terms of the cell behavior and response. Nevertheless, since this study focuses on the effect of different loading conditions on the cells, the relative differences in cell response observed between the different magnitudes of strains and frequencies regimes are considered reliable and relevant.

Thirdly, although comprehensive gene expression analyses have been performed in this work, which not only included ECM and tenogenic genes but also other mesoderm lineage markers belonging to the osteogenic, chondrogenic, and myogenic tissues, the translation of the mRNA expression into protein synthesis was not evaluated. This would be important to evaluate in order to determine whether the mechanostimulated scaffolds are able to elicit a functional response from the ASCs and contribute to deposition of new matrix.

## 5. Conclusion

In conclusion, the combined results of the ASC-encapsulated collagen scaffolds subjected to mechanical stimulation at 2% strain and 0.1 Hz frequency indicate key features: (a) there is a definitive change in the ASC morphology with the rounded cells resembling more like tendon fibroblasts, with their elongated shape and the cytoplasmic extensions, (b) the scaffold matrix shows distinct organization with the directionality of collagen fibers being parallel to the axis of load application, (c) the gene expression data demonstrates significant increases in ECM and tendon-related genes, and (d) no cross-differentiation potential of ASCs to osteogenic, chondrogenic, or myogenic lineage is observed giving rise to pure tenogenic differentiation. Thus, 2% strain at 0.1 Hz frequency is identified to be the appropriate uniaxial mechanical loading strain and frequency to induce tenogenic differentiation of ASCs for tendon tissue engineering. This study primarily uses gene expression analyses to determine the role of different physiological mechanical strains and frequencies in eliciting a tenogenic response from ASCs. Further work is required to evaluate the protein expression profile exhibited by ASCs, and the signaling pathways that drive the mechanical loading-induced ASC tenogenesis within 3D collagen scaffolds at the identified uniaxial tensile strain of 2% with 0.1 Hz frequency.

## Figures and Tables

**Table 1:** Forward and reverse primers used for real-time PCR.

Gene	Forward primer	Reverse primer	Ref
GAPDH	5′ AGAAGGCTGGGGCTGATTTG 3′	5′ AGGGCCCATCCACAGTCTTC 3′	[33]
COL I	5′ GGCTCCTGCTCCTCTTAGCG 3′	5′ CATGGTACCTGAGGCCGTTC 3′	[25]
COL III	5′ CAGCGGTTCTCCAGGCAAGG 3′	5′ CTCCAGTGATCCCAGCAATCC 3′	[25]
DCN	5′ CGCCTCATCTGAGGGAGCTT 3′	5′ TACTGGACCGGGTTGCTGAA 3′	[25]
ACAN	5′ CACTGTTACCGCCACTTCCC 3′	5′ ACCAGCGGAAGTCCCCTTCG 3′	[34]
TCN	5′ GGTGGATGGATTGTGTTCCTGAGA 3′	5′ CTGTGTCCTTGTCAAAGGTGGAGA 3′	[25]
SCX	5′ ACACCCAGCCCAAACAGA 3′	5′ GCGGTCCTTGCTCAACTTTC 3′	[25]
TNMD	5′ CCATGCTGGATGAGAGAGGT 3′	5′ CTCGTCCTCCTTGGTAGCAG 3′	[34]
RUNX2	5′ CAACCACAGAACCACAAGTGC 3′	5′ TGTTTGATGCCATAGTCCCTCC 3′	[25]
ALP	5′ GATCTTCTTTCTCCTTTGCCTGG 3′	5′ TGTTTGCAGTGGTGGTTCTGGCA 3′	[26]
COL II	5′ GGCAATAGCAGGTTCACGTACA 3′	5′ CGATAACAGTCTTGCCCCACTT 3′	[35]
Sox 9	5′ CACACAGCTCACTCGACCTTG 3′	5′ TTCGGTTATTTTTAGGATCATCTCG 3′	[26]
MyoD	5′ GCAGGTGTAACCGTAACC 3′	5′ ACGTACAAATTCCCTGTAGC 3′	[36]
MYOG	5′ GCCACAGATGCCACTACTTC 3′	5′ CAACTTCAGCACAGGAGACC 3′	[36]
